# Expression and Immune Responses to MAGE Antigens Predict Survival in Epithelial Ovarian Cancer

**DOI:** 10.1371/journal.pone.0104099

**Published:** 2014-08-07

**Authors:** Sayeema Daudi, Kevin H. Eng, Paulette Mhawech-Fauceglia, Carl Morrison, Anthony Miliotto, Amy Beck, Junko Matsuzaki, Takemasa Tsuji, Adrienne Groman, Sacha Gnjatic, Guillo Spagnoli, Shashikant Lele, Kunle Odunsi

**Affiliations:** 1 Department of Gynecologic Oncology, Roswell Park Cancer Institute, Buffalo, New York, United States of America; 2 Department of Biostatisticsm, Roswell Park Cancer Institute, Buffalo, New York, United States of America; 3 Department of Pathology, University Southern California, Los Angeles, California, United States of America; 4 Department of Pathology, Roswell Park Cancer Institute, Buffalo, New York, United States of America; 5 Center for Immunotherapy, Roswell Park Cancer Institute, Buffalo, New York, United States of America; 6 Department of Immunology, Roswell Park Cancer Institute, Buffalo, New York, United States of America; 7 Department of Medicine, Mount Sinai Hospital, New York, New York, United States of America; 8 Department of Biomedicine, University Hospital Basel, Basel, Switzerland; King's College London, United Kingdom

## Abstract

The MAGE cancer-testis antigens (CTA) are attractive candidates for immunotherapy. The aim of this study was to determine the frequency of expression, humoral immunity and prognostic significance of MAGE CTA in human epithelial ovarian cancer (EOC). mRNA or protein expression frequencies were determined for MAGE-A1, -A3, -A4, -A10 and -C1 (CT7) in tissue samples obtained from 400 patients with EOC. The presence of autologous antibodies against the MAGE antigens was determined from 285 serum samples. The relationships between MAGE expression, humoral immunity to MAGE antigens, and clinico-pathologic characteristics were studied. The individual frequencies of expression were as follows: A1: 15% (42/281), A3: 36% (131/390), A4: 47% (186/399), A10: 52% (204/395), C1: 16% (42/267). Strong concordant expression was noted with MAGE-A1:–A4, MAGE-A1:–C1 and MAGE-A4:–A10 (*p*<0.0005). Expression of MAGE-A1 or -A10 antigens resulted in poor progression free survival (PFS) (OR 1.44, CI 1.01–2.04, *p* = 0.044 and OR 1.3, CI 1.03–1.64, *p* = 0.03, respectively); whereas, MAGE-C1 expression was associated with improved PFS (OR 0.62, CI 0.42–0.92, *p* = 0.016). The improved PFS observed for MAGE-C1 expression, was diminished by co-expression of MAGE-A1 or -A10. Spontaneous humoral immunity to the MAGE antigens was present in 9% (27/285) of patients, and this predicted poor overall survival (log-rank test *p* = 0.0137). These findings indicate that MAGE-A1, MAGE-A4, MAGE-A3, and MAGE-A10 are priority attractive targets for polyvalent immunotherapy in ovarian cancer patients.

## Introduction

Epithelial ovarian cancer (EOC) represents the most lethal gynecologic malignancy in women. Despite considerable efforts directed at early detection and improving response rates, the majority of women present with disseminated disease at initial diagnosis, carry an unacceptable relapse rate of approximately 85% and a 5-year overall survival of 20–30% [1], [2]. Consequently, targeted treatment strategies, such as immunotherapy will be required to improve the clinical outcome of ovarian cancer patients.

The development of successful immunotherapy requires the characterization of tumor-associated antigens (TAA) that are commonly expressed in ovarian tumors, with a restricted expression pattern in normal tissues. Moreover, the ideal antigen should exhibit a high frequency of expression in cancer and evidence of immunogenicity. Candidate TAA are often identified in patients with strong cellular and/or humoral immune responses that indicate robust inherent immunogenicity to these antigens [3]–[5].

The cancer testis antigens (CTA) are a subclass of TAA encoded by approximately 140 genes. Despite their poorly characterized biologic function, expression of these antigens are known to be restricted in immune privileged sites such as the testes, placenta and fetal ovary, but not in other normal tissues. Abnormal expression of these germ-line genes in malignant tumors may reflect the activation of a silenced “gametogenic program”, which ultimately leads to tumor progression and broad immunogenicity [6]. The immunogenicity of CTA has led to the widespread development of cancer vaccines targeting these antigens in many solid tumors. Within this large class of TAA, melanoma-associated antigens (MAGE) have emerged as promising candidates for cancer immunotherapy [7]–[9].

More than 30 cancer testis (CT) genes have been reported as members of multi-gene families that are organized into gene clusters on chromosome X (CT-X antigens). The CT gene clusters are located between Xq24 and Xq28 and include gene families such as MAGE and NY-ESO-1 [10]. Type I MAGE gene clusters are the most extensively characterized and include the MAGE-A, MAGE-B and MAGE-C families. The MAGE-A proteins are encoded by 12 different MAGE-A gene family members (MAGE-A1 to MAGE-A12) and are defined by a conserved 165–171 amino acid base, called the MAGE homology domain (MHD). The MHD corresponds to the only region of shared amino acids by all of the MAGE-A family members. MAGE-C1/CT7 is structurally different from MAGE-A family, with a protein product of 1142 amino acids (vs.<400 residues for the MAGE-A proteins) that contains a tandem repeat sequence that is absent in MAGE-A [11].

In the present study, we have analyzed the expression and immunogenicity of a panel of five MAGE CTA in a large cohort of ovarian cancer patients. In addition, we have examined the relationship between coordinate expression of MAGE genes and clinico-pathologic outcomes.

## Materials and Methods

### Patients and Specimens

Formalin-fixed paraffin-embedded (FFPE; for immunohistochemistry) and snap-frozen tissue specimens (for reverse transcriptase-PCR) were obtained from 400 patients undergoing cytoreductive surgery for ovarian, primary peritoneal and fallopian tube cancer at the Roswell Park Cancer Institute (Buffalo, NY) between 1992 and 2008. We identify, and refer to, these three cancers as EOC due to their common origin in the mullerian epithelium. All tissue specimens were collected under an approved protocol from the institutional review board (IRB) of Roswell Park Cancer Institute. Patients signed an IRB approved written informed consent, and these were filed in the IRB office. All pathology specimens were reviewed in our institution, and the histopathologic subtype of the tumors was classified according to the guidelines of the World Health Organization (WHO) [12], [13]. The stage and grade of the tumors were assessed according to the International Federation of Gynecology and Obstetrics (FIGO) [14], [15]. In a subset of the patients, serum samples were collected at diagnosis. The medical records of these patients were retrospectively reviewed under an approved institutional review board protocol. The review included outpatient and inpatient treatment. Study outcomes included overall survival (OS) and progression free survival (PFS). Both survival criteria were measured from the time of diagnosis. Recurrence was defined via objective criteria as all therapy was given in the adjuvant setting. The duration of OS was the interval between diagnosis and death. PFS represented the interval between diagnosis to disease progression, recurrence or death. The observation time was the interval between diagnosis and last contact (death or last follow-up). Data were censored at the last follow-up for patients with no evidence of recurrence, progression, or death.

### Total Tissue RNA Isolation

Total tissue RNA was isolated from frozen tumor tissues by use of the TRI Reagent (Molecular Research Center Inc, Cincinnati, OH, USA) according to the manufacturer's protocol. Potentially contaminating DNA was removed by treating with RNase-free DNase I (Boehringer-Mannheim, Mannheim, Germany), followed by phenol/chroloform extraction. RNA was dissolved in RNase-free H_2_O. The resulting RNA concentration was measured spectrophotometrically (DU500 Spectrophotometer, Beckman Coulter, Fulleron, CA, USA), and the quality of the RNAs was checked by electrophoresis on 1.5% agarose gel.

### Reverse Transcriptase-PCR Analysis of MAGE-A1, MAGE-A3, MAGE-A4, MAGE-A10 and MAGE-C1 Expression

Two micrograms of each RNA sample were used to generate cDNA with the Ready-To-Go Reverse Transcriptase-PCR (rt-PCR) beads (GE Healthcare, Buckinghamshire, UK). RNA from normal testicular tissue (Clontech, Mountain View, CA) was used as a positive control. PCR was subsequently performed to study the expression of MAGE-A1, -A3, -A4, -A10 and -C1 in 305 patients with EOC. Glyceraldehyde-3-phosphodehydrogenase (GAPDH) primers were used as a test for RNA integrity. **Table S1** lists the primer sequences for each gene and its respective amplicon length. The amplification conditions for all gene products was 5 min at 95°C, followed by 35 cycles that consisted of 1 min at 95°C, 1 min at 60°C, and 1 min at 72°C. These cycles were followed by a 6-min elongation step at 72°C (BioRad iCycler, BioRad Laboratories, Hercules, CA, USA). The PCR products were separated over a 1.5% agarose gel and visualized with ethidium bromide on an ultraviolet transilluminator (IS-4400 ChemiImager, Alpha Innotech, San Leandro, CA). The intensities of the PCR products were heterogeneous, and some specimens yielded only faint amplicon bands. These were scored positive only if the result could be reproduced by a repeated RNA extraction and specific rt-PCR from the same tumor specimen. Cases with very low transcript levels that were not reproducibly positive were not regarded as positive. PCR product bands were excised from the agarose gel and the associated DNA was isolated with the QIAquick Gel Extraction Kit (Qiagen, Valencia, CA, USA). DNA samples were submitted for sequencing to verify the PCR product.

### Immunohistochemical Analysis of MAGE-A3, MAGE-A4 and MAGE-A10 Expression

Immunohistochemical (IHC) analysis was performed using FFPE tissues from 304 patients on tissue microarrays (TMA). TMA were constructed using 0.6 mm FFPE tissue cores punched from each donor block. To overcome tumor heterogeneity, three representative cores were selected from each tumor. The 4 µm-thick tissue cores were deparaffinized and pretreated with a specific antigen retrieval solution (Dakocytomation, Carpenteria, CA) over 20 minutes. Slides were cooled for 20 minutes and then treated in 3% H_2_O_2_ to quench endogenous peroxidase activity. The TMA slides were then incubated with a serum-free protein block (Dakocytomation, Carpenteria, CA) for 30 minutes. **Table S2** lists the antibodies and IHC conditions. MAGE-A3 rabbit anti-human polyclonal antibody (LS-B4662, Lifespan Biosciences, Inc.) was commercially acquired. Anti-MAGE-A4 mAb (clone: 57b) and anti-MAGE-A10 (clone: A3) hybridoma supernatants were produced at the University Hospital Basel (Basel, Switzerland) [16], [17]. Rabbit IgG or mouse IgG1 (Sigma, St. Louis, MO) was used as the negative isotype matched control. Labeled streptavidin biotin (LSAB+) reagents (Dakocytomation, Carpenteria, CA) were used according to the manufacturer's instructions followed by a 3,3′-diaminobenzidine (DAB)+ (Dakocytomation, Carpenteria, CA) incubation. Sections were counterstained with hematoxylin. A cut-off of 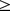
5% positive tumor cells was used to define positive expression.

### Measurement of serum antibody by ELISA

Serologic analysis of humoral immune responses was performed as previously described [18]. 285 serum samples from a subset of the patients were analyzed by ELISA for seroreactivity to bacterially produced full-length recombinant proteins MAGE-A1, -A3, -A4, -A10 or -C1. As a negative control antigen, recombinant dihydrofolate reductase protein was prepared and used in each assay. Serum was diluted serially from 1∶100 – 1∶100,000 and added to low-volume 96-well plates (Corning) coated overnight at 4°C with 1 µg/mL antigen in 25 µl and blocked for 2 h at room temperature with PBS containing 5% nonfat milk. After overnight incubation, plates were extensively washed with PBS containing 0.2% Tween 20 and rinsed with PBS (BioTek ELx405 automated washer). Serum IgG bound to antigens was detected with goat anti-human IgG antibodies conjugated to alkaline phosphatase (Southern Biotech). Following addition of ATTOPHOS substrate (Fisher Scientific), fluorescent signal was measured using a Cytofluor Series 4000 fluorescence reader (PerSeptive Biosystems). A reciprocal titer was calculated for each plasma sample as the maximal dilution still significantly reacting to a specific antigen. Specificity was determined by comparing seroreactivity among the various antigens tested. In each assay, sera of patients with known reactivity were used as controls. A positive result was defined as reciprocal titers >100.

### Statistical Analysis

All statistical analyses were generated using SAS software (SAS System Copyright 2002 SAS Institute Inc. v.9.2) and the R 2.15.3 statistical computing language. A nominal significance level of 0.05 was used in all testing. Using a 2×2 contingency table, the level of concordance among the various MAGE gene expression profiles was determined. The distributions of MAGE-A1, -A3, -A4, -A10 and -C1 expression and clinical outcome were analyzed by the Wilcoxon Rank Sum Test or Pearson Chi Square Test. Multivariate analysis for independent predictors of survival were tested using the Cox proportional hazard model [19]. Estimated survival distributions were calculated by the method of Kaplan and Meier [20], and tests of significance with respect to survival distributions were based on the log-rank test. Relative prognosis was summarized using estimates and 95% confidence limits for the hazard ratio (HR). No adjustments were made for multiple comparisons. A phylogenetic tree is constructed by a Manhattan distance, coding expression as zero (absent) and one (present), and the standard neighbor joining algorithm.

## Results

### Study Population

A total of 400 tissues from patients with ovarian, primary peritoneal and fallopian tube cancers were investigated by rt-PCR and IHC. The characteristics of the patients in this study are presented in **Table 1**. The median age of the patient sample was 63 (range: 21–93), with a median duration of follow-up of 35 months (range: 1–176 months). As expected, the majority of patients presented with advanced stage disease (82%), poorly differentiated tumors (74%) and with serous histology (64%). Platinum sensitive disease was demonstrated in 182 of the 400 patients (46%), with 116 patients having platinum resistance (29%), and 16 patients with a platinum refractory response (4%). The median OS for all patients was 40 months (range: 0–173), whereas the median PFS was 12 months (range: 0–160).

**Table 1:** Patient characteristics.

Patients eligible for analysis	400
**Age** Median (Range) (Years)	63 (21–93)
**PFS** Median (Range) (Months)	12 (0.1–160)
**OS** Median (Range) (Months)	40 (0.1–173)
**FU** Median (Range) (Months)	35 (0.7–176)
**Primary Site**	
Fallopian Tube	8 (2%)
Ovary	339 (84%)
Primary Peritoneal	53 (14%)
**FIGO Stage**	
Early Stage	69 (18%)
Advanced Stage	323 (82%)
**Histology**	
Clear Cell	21 (5%)
Endometrioid	18 (4.5%)
Mucinous	18 (4.5%)
Serous	254 (64%)
Other^a^	89 (22%)
**Grade**	
1	29 (7%)
2/3	353 (88%)
**Debulking Status**	
Optimal	301 (75%)
Suboptimal	90 (23%)
Unknown	9 (2%)
**Platinum Status**	
Sensitive	182 (46%)
Resistant / Refractory	132 (33%)
Unknown	86 (21%)
**Recurrences**	
No Recurrence	67 (17%)
Recurrence / Persistent Disease	162 (41%)
No Disease Free Interval	98 (25%)
Unknown	65 (17%)
**Current Status**	
Alive No Evidence of Disease	84 (22%)
Alive with Disease	37 (10%)
Dead	270 (68%)

PFS  =  Progression Free Survival. OS  =  Overall Survival. FU  =  Follow Up.

a Other Histology includes Borderline, Carcinosarcoma, Granulosa Cell, Mixed, Sertoli Leydig, Sex Cord Stromal, Signet Ring Cell, Small Cell Type, Transitional, Anaplastic, Undifferentiated and Poorly Differentiated tumors.

b Numbers do not add up to 100% due to unknown categories.

### Expression of MAGE-A1, MAGE-A3, MAGE-A4, MAGE-A10 and MAGE-C1 in Ovarian Cancer

Expression of MAGE antigens was evaluated by both rt-PCR and IHC for the majority of patients from whom appropriate samples were available. The expression of MAGE-A4 and MAGE-A10 was detected concordantly by both methods (r = 0.31, OR = 3.88, p<0.001; r = 0.14, OR = 2.00, p<.001 respectively) but MAGE-A3 results were not reproducibly concordant (r = −0.06, OR = 0.86, p = 0.9198) due to a low rate of detection (**Table S3**). Unless otherwise stated, we proceeded by classifying tissues as antigen positive if they were identified by rt-PCR or IHC (**Table 2**). Consequently, when considering all 400 tissues samples analyzed by rt-PCR or IHC in this study, the frequencies for MAGE-A3, -A4 and –A10 expression by either method were 36% (131/390), 47% (186/399) and 52% (204/395) of the tissues, respectively (**Table 2**). MAGE-A1 and -C1 demonstrated the lowest frequency of expression at 15% (42/281) and 16% (42/267), respectively. **Table S3** is a summary of the frequency of MAGE mRNA and protein expression in tumor specimens from the EOC patients.

**Table 2:** Serum antibody and co-expression status for MAGE antigens in ovarian cancer.

MAGE Antigen	A1	A3	A4	A10	C1	Any A
**Autoantibody (ELISA)**	10/285 (4%)	12/285 (4%)	7/115 (6%)	6/86 (7%)	10/93 (11%)	27/285 (9%)
**Expression (rt-PCR or IHC)**	42/281 (15%)	131/390 (36%)	186/399 (47%)	204/395 (52%)	42/267 (16%)	310/400 (78%)

MAGE Co-expression	A1	A3	A4	A10	C1
**MAGE-A1**		23/281 (8%)	31/281 (11%)	23/277 (8%)	12/258 (5%)
**MAGE-A3**			70/289 (18%)	81/386 (21%)	18/267 (7%)
**MAGE-A4**				114/394 (29%)	24/267 (9%)
**MAGE-A10**					25/264 (10%)

A total of 400 patients were studied for MAGE expression. A subset of 285 patients were studied for anti-MAGE autoantibody. The numerator represents the number of antigen positive tumors or serology. The denominator represents the total number of successful assays for each antigen. Antigen specific numbers vary due to assay viability. Percentages represent the frequency of MAGE expression or MAGE-specific antibody.

**Figure 1:**
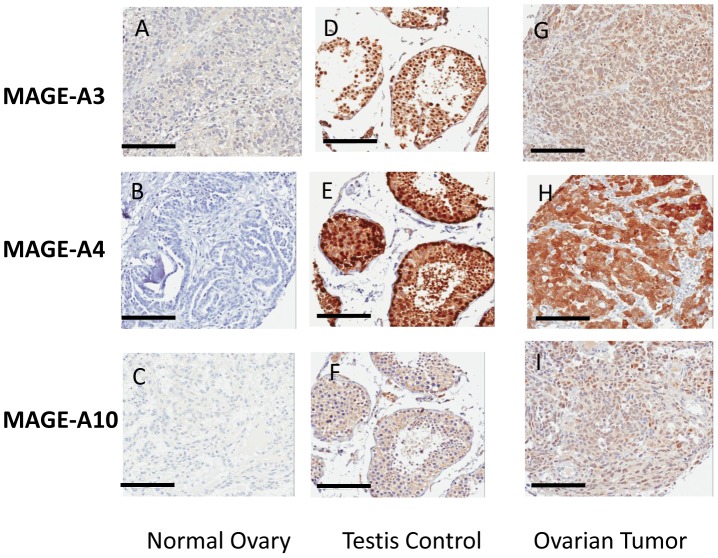
A–I: Immunohistochemical staining for MAGE. Specimens were stained with polyclonal antibody for MAGE-A3 (X20), clones 57b and A3 hybridoma supernatants for MAGE-A4 and MAGE-A10, respectively (x15). Specimens from the normal ovary and testis were used as negative and positive controls, respectively. A–C: Staining of the normal ovary showing no reactivity. D–F: Staining of the testis showing seminiferous tubules with strong intratubular staining, and absent non-specific reactivity. G–I: Staining of ovarian tumor demonstrating strong cytoplasmic and/or nuclear staining patterns.

MAGE-A3, -A4 and -A10 exhibited no immunostaining in normal tissues (**Figs.1A–C**) but intense immunostaining in testis (**Figs.1D–F**). The staining pattern was cytoplasmic and nuclear for MAGE-A3 and A-4, and diffuse cytoplasmic staining for MAGE-A10 (**Figs.1G–I**).

### Co-expression of MAGE-A and MAGE-C1 in Ovarian Cancer

**Figure 2:**
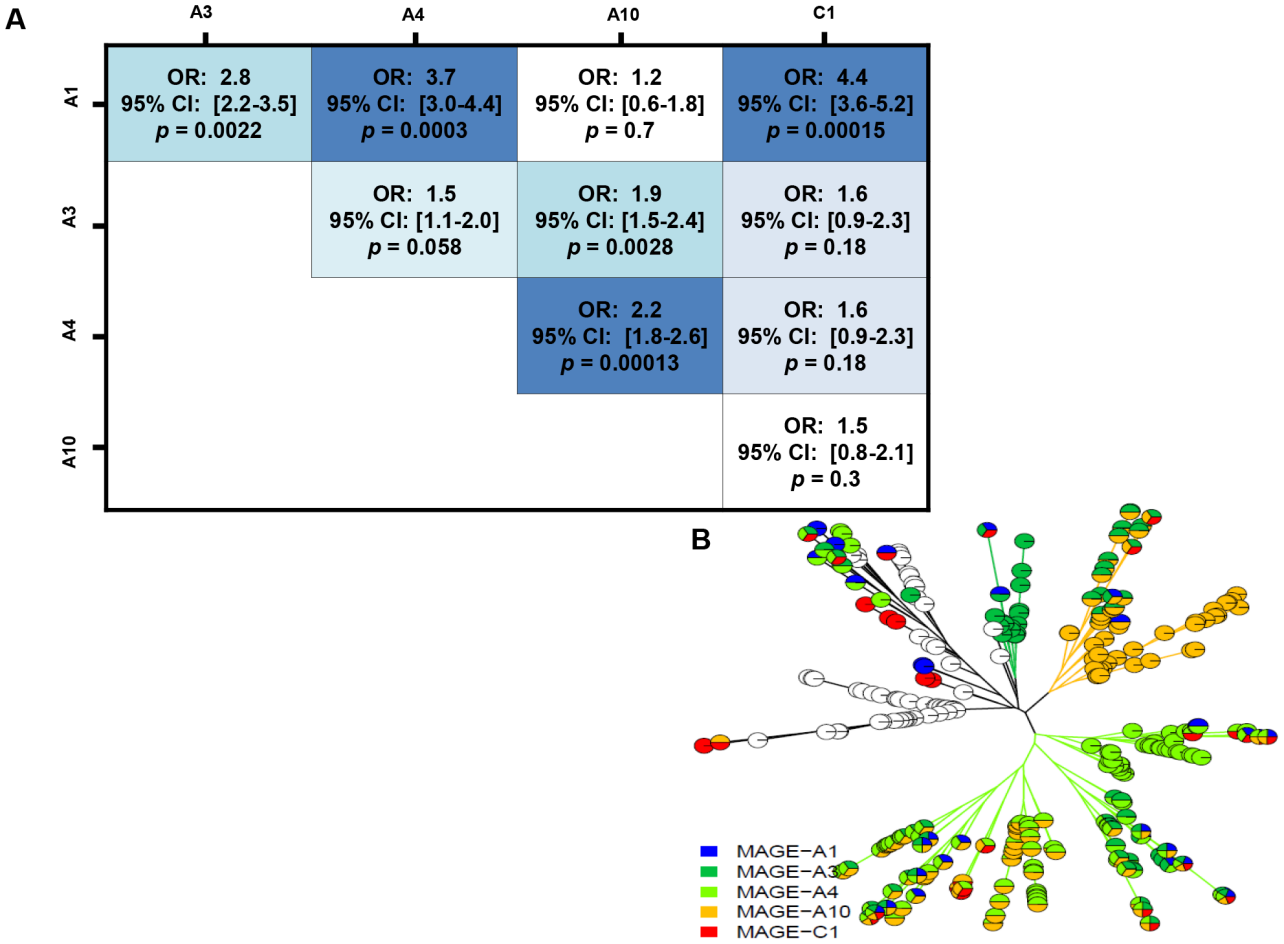
A–B: Co- expression of MAGE antigens in ovarian cancer. (A) MAGE-A1 is co-expressed with –A3 or –A4 or –C1. MAGE-A3 is co-expressed with –A10. MAGE-A4 is co-expressed with MAGE-A10. The darker color intensity represents a stronger significance. The strongest associations are between MAGE-A1 to –A4, MAGE-A1 to –C1 and MAGE-A1 to –A3. Odds ratios (OR) greater than 1 imply the antigens tend to appear together. (B) Phylogenetic tree for MAGE expression. Each leaf ending in a pie chart symbolizes a person.

The frequencies of co-expression of the MAGE antigens are shown in **Table 2**. The highest frequency of co-expression was observed for -A4:-A10 (29%). While they are both comparatively rare, the MAGE-C family antigen (-C1) tended to be co-expressed with MAGE-A1 [OR 4.4, p = 0.00015, CI 3.6–5.2] and to be expressed independently with the other MAGE-A family antigens (**Figure 2a**). MAGE-A family antigens -A1:-A4 [OR 3.7, p = 0.0003, 3.0-4.4] and –A1:-A3 [OR 2.8 p = 0.0022, CI 2.2–3.5] have the strongest co-expression; nearly every pair is associated except for –A1:-A10 (OR = 1.2, p = 0.7 CI 0.6–1.8) which suggests they are expressed independently. To characterize the multivariate pattern of MAGE expression, the phylogenetic tree in **Figure 2b** was created, whereby each leaf ending in a pie chart symbolizes a person or set of people. There are two distinct patterns of expression that are observed. The MAGE-A4 gene directs a major pattern of expression (lower right hand clades), and then later develops into MAGE-A3 and -A10 expression in this patient population. The second unique expression pattern consists of an independent expression of MAGE-A3 and -A10, which then develops into -A4 (upper right hand clades). In stark contrast, MAGE-C1 expression rarely appears alone and emerges sporadically throughout the phylogenetic tree. Similarly, MAGE-A1 appears infrequently and rarely in the MAGE-A10 clade. These observations translate into strong clinical implications within this large study cohort.

### Correlation of MAGE Antigen Expression with Clinical Outcome

The relationship between MAGE expression and clinic-pathologic parameters was investigated. We found that MAGE-A3 and A4 did not have individual prognostic effects. Consequently, we focused further analysis on MAGE-A1, MAGE-A10, MAGE-C1 and other known clinico-pathologic prognostic factors in ovarian cancer.

**Figure 3:**
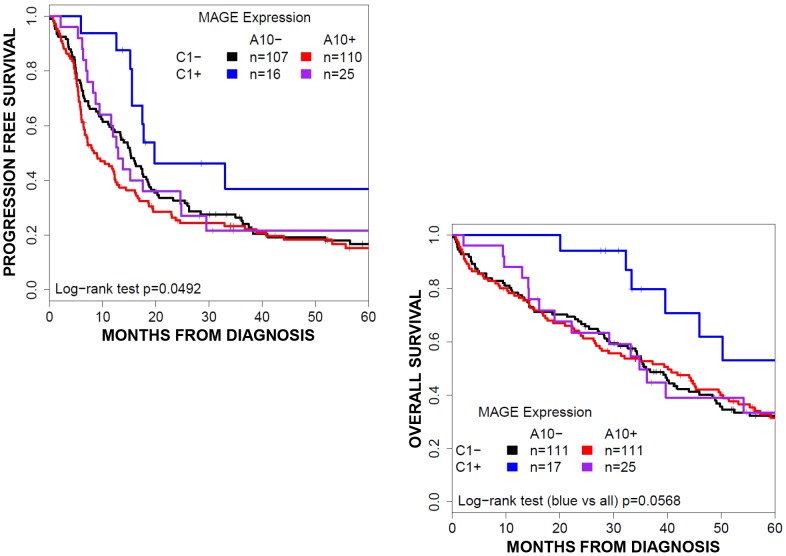
Survival by MAGE expression. Overall survival curves for patient groups based on MAGE-A10 and –C1 expression. MAGE-C1 expression predicts an improved progression free survival and a trend towards improved overall survival. Expression of MAGE-A10 dampens survival outcomes to the degree of patients with negative MAGE expression.

Alone, MAGE-A10 expression (204/395, 52%) was associated with worse clinical outcome (median PFS 8.8[7.2–12.3] vs. 15.5[13.6–18.4] months, *p* = 0.009; OS 37.8[28.2–45.0] vs. 45.0[39.8–52.2] months, p = 0.0781). Co-expression with MAGE-C1 (25/204, 12%) reversed this trend for PFS but not OS: A10(+)/C1(−) patients had median PFS of 7.9 months (7.0–12.1) versus A10(+)/C1(+) 13.1 months (CI9.5–29.9). Stratified into four groups based on A10/C1 expression pattern, this pattern holds (log-rank test p = 0.0133) and suggests that A10(−)/C1(+) expression may be protective (median PFS 20 [15.7-NA] months) (**Fig. 3**).

Expression patterns were not jointly associated with age (p = 0.900), stage (p = 0.1373), grade (p = 0.4532), histology (p = 0.737), primary site (p = 0744) or debulking status (p = 0.112) (**Table 3**). MAGE-C1 expression was associated with platinum sensitive disease (28/38, 74% vs. 70/142, 49%; p = 0.009) and clinical response (30/38, 79% vs. 92/164, 56%, p = 0.015).

**Table 3:** Patient Characteristics by MAGE Expression.

Clinical and Pathologic Features	MAGE-A10(-) MAGE-C1(-)	MAGE-A10(-) MAGE-C1(+)	MAGE-A10(+) MAGE-C1(-)	MAGE-A10(+) MAGE-C1(+)	*p* value
All tumors (n)	193/258 (75%)	29/258 (11%)	24/258 (9%)	12/258 (5%)	
Age (Years)	63 (21–89)	66 (37–84)	69 (34–91)	62 (35–86)	0.9
PFS (95% CI)	15 (15.7–NA)	20 (15.7–NA)	8 (7–12.2)	13 (10–30)	**0.005**
OS (95% CI)	43 (36–52)	68 (40–NA)	38 (28–45)	35 (23–NA)	0.192
**FIGO Stage**					0.1373
Early Stage (I–II)	35	6	0	2	
Late Stage (III–)	154	23	24	10	
**Tumor Grade**					0.4532
1	18	3	1	0	
2/3	171	25	23	12	
**Histology**					0.737
Clear Cell	12	3	0	0	
Endometrioid	7	1	1	1	
Mucinous	12	0	1	0	
Serous	115	19	17	9	
Other	47	5	4	2	
**Primary Site**					0.744
Ovarian	159	25	17	11	
Primary Peritoneal	29	3	6	1	
Fallopian Tube	5	1	1	0	
**Debulking Status**					0.112
Optimal	147	25	16	11	
Suboptimal	38	4	8	1	
**Platinum Status**					**0.008**
Sensitive	72	21	7	7	
Resistant / Refractory	70	6	14	4	
**Clinical Response**					**0.018**
Complete Response	84	21	8	9	
Persistent Disease	59	6	13	2	

PFS  =  Months Progression Free Survival. OS  =  Months Overall Survival. NA  =  upper limit not estimated.

aOther Histology includes Borderline, Carcinosarcoma, Granulosa Cell, Mixed, Sertoli Leydig, Sex Cord Stromal, Signet Ring Cell, Small Cell Type, Transitional, Anaplastic, Undifferentiated and Poorly Differentiated tumors.

Pvalues are for any difference among the columns.

Although we focused on MAGE-A10, the less prevalent MAGE-A1 (42/281, 15%) was also mildly associated with poorer survival outcomes (median PFS 10.3[6.2–15.0] vs 12.8[10.5–15.7] months *p* = 0.043, OS 38.7[20.3–78.4] vs. 41.3[36.5–46.6] months p = 0.607). For PFS, MAGE-A1 co-expression with A10 is redundant (stratified log-rank test p = 0.57) however A1(+)/A10(−) patients have slightly poorer prognosis (median PFS 12.7 [5.3–36.6] vs. 16.3 [14.1–19.4] months, p = 0.0187). To model all three antigens jointly, we recommend considering the expression of either the A1 or A10 antigen along with the expression of MAGE-C1; the effect is not significantly different from the stratification presented in **Table 3**.

### Correlation of Antibody Response to the MAGE Antigen with Clinical Outcome

**Figure 4:**
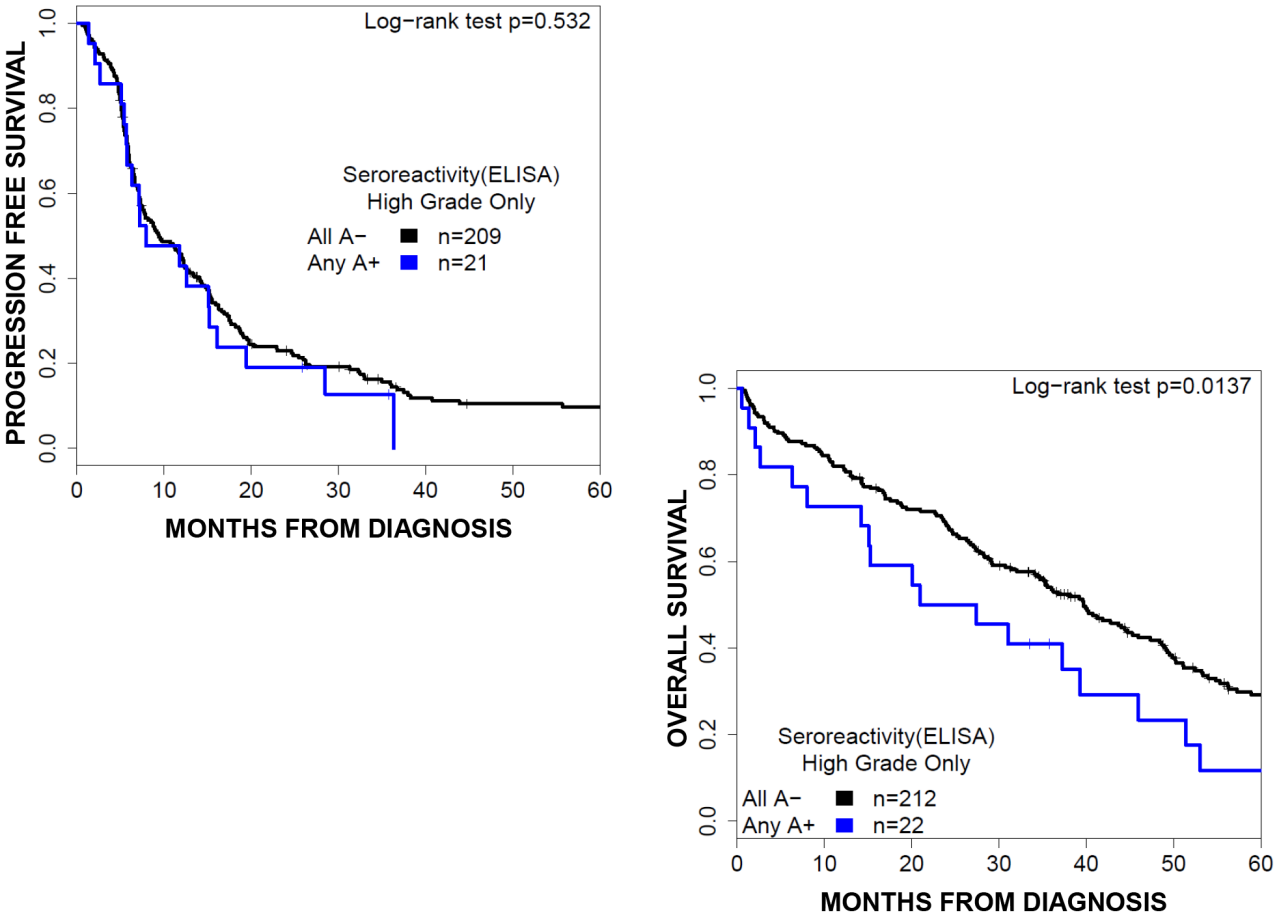
Survival by MAGE serology. Overall survival curves for patients groups based on the presence of anti-MAGE autoantibody. Humoral response to any MAGE antigen predicts poor overall survival, and no significant association with progression free survival.

ELISA for serum MAGE antigen-specific antibodies was performed on serum samples obtained at diagnosis from 285 of the 400 EOC patients (**Table 2**). Spontaneously induced humoral response to at least one MAGE-A antigen was observed in 9% (27/285) of patients of which 85% (23/27) also expressed a MAGE-A antigen. The serologic response to any MAGE antigen was equally distributed among all clinico-pathologic parameters (**Table 4**). The presence of humoral immune response to any of the MAGE antigens predicted a worse overall survival (median PFS 12.3[6.4–19.7] vs. 13.5[10.9–16.5] months, p = 0.231; median OS 27.8[17.3–52.2] vs 45.4[40.2–51.9] months, p = 0.002) (**Figure 4**).

**Table 4:** Patient Characteristics by MAGE Serology.

Clinical and Pathologic Features	All MAGE-A (−)	Any MAGE-A (+)	*p* value
All tumors (n)	258/285 (91%)	27/285 (9%)	
Age [Median (range)] (Years)	63 (21–89)	69 (43–89)	0.017
PFS (95% CI)	14 (11–17)	12 (6–20)	**0.231**
OS (95% CI)	45 (40–52)	28 (17–52)	**0.002**
**FIGO Stage**			0.828
Early Stage (I–II)	43	5	
Late Stage (III–IV)	212	22	
**Tumor Grade**			0.428
1	20	3	
2/3	233	24	
**Histology**			0.241
Clear Cell	18	0	
Endometrioid	9	2	
Mucinous	12	2	
Serous	165	13	
Other	54	10	
**Primary Site**			0.017
Ovarian	215	22	
Primary Peritoneal	38	5	
Fallopian Tube	5	0	
**Residual Tumor at Cytoreduction**			0.765
Optimal	193	22	
Suboptimal	58	5	
**Platinum Status**			0.616
Sensitive	110	12	
Resistant / Refractory	104	8	
**Clinical Response**			0.877
Complete Response	124	12	
Persistent Disease	89	8	

PFS  =  Progression Free Survival. OS  =  Overall Survival. FU  =  Follow Up.

a Other Histology includes Borderline, Carcinosarcoma, Granulosa Cell, Mixed, Sertoli Leydig, Sex Cord Stromal, Signet Ring Cell, Small Cell Type, Transitional, Anaplastic, Undifferentiated and Poorly Differentiated tumors.

## Discussion

Immunotherapy is a promising approach to improve survival rates and clinical outcomes in ovarian cancer patients [21]–[23]. Among the possible tumor antigen targets, CTA are considered as the most promising candidates for the development of anti-cancer vaccines. To assess the utility of the MAGE family CTA as targets for specific immunotherapy in EOC, the present comprehensive analysis was undertaken on a large panel of ovarian tumors. Our results indicate aberrant expression of MAGE-A1, MAGE-A3, MAGE-A4, MAGE-A10 and MAGE-C1 in 15%, 36%, 47%, 52% and 16% of EOC specimens, respectively. In addition, we found that considering any of these MAGE antigens, approximately 78% showed expression of at least one of these five CT antigens. Moreover, MAGE-A1 and MAGE-A10 expression were associated with poor clinical outcome, while MAGE-C1/CT7 was associated with improved survival.

The frequency of MAGE expression in EOC that we report is generally consistent with that reported for the majority of other tumors, except for melanoma and non-small cell lung cancer [16], [24]. Expression of MAGE-A3 mRNA has been found in 10–40% of several tumor types, including bladder [25], breast [26] and multiple myeloma [27]. With respect to EOC, the frequency of MAGE-A1 and -A3 expression that we report is similar to that reported in previous studies, with the exception of Zhang *et al* who found a 54% and 37% expression in ovarian cancer tissues, respectively [28]–[30]. This may reflect differences in the study populations, as there were far more early stage tumors than our study group. In a previous study, the expression of MAGE-A4 adversely correlated with survival or indirectly to established prognostic factors in ovarian cancer [31]. Consistent with these studies, our findings demonstrate a clear association for MAGE expression and prognosis. In this regard, while MAGE-A1 and MAGE-A10 expression were associated with poor clinical outcome, MAGE-C1 was associated with improved survival. These contrasting survival findings among the different MAGE antigen families raise many essential questions regarding the role of the MAGE genes in tumorigenesis, invasion and metastasis in EOC.

In general, MAGE antigens are more often expressed in patients with advanced disease and poor outcome, indicating that their expression might contribute to tumorigenesis [26], [32]–[37]. Several studies have demonstrated that MAGE proteins are critical to cell survival, increasing the tumorigenic properties of cells and therefore, may actively contribute to the development of malignancies [38]–[40]. Moreover, since CT antigen expression has been associated with tumorigenic transformation of cancer stem cells [41], it is possible that MAGE-A1 and MAGE-A10 expressing ovarian cancer cells represent a population with self-renewing stem cell properties, and therefore more resistant to immune elimination or chemotherapy.

In contrast to MAGE-A1 and MAGE-A10, the possible mechanism(s) by which expression of MAGE-C1/CT7 confers a survival benefit is less clear. In comparing the protein structure of the MAGE family members, while the first and second domains are highly conserved among the MAGE-A and –C family members, the MAGE-C1 protein carries a unique feature. In addition to a 275-amino acid MAGE-homologous segment on its C-terminus, MAGE-C1/CT-7 has an 867-amino acid region composed of three types of tandem repeats in its N-terminus [11]. This region may be of significant importance as the repetitive protein sequence may shape the epitopes presented for immune recognition of MAGEC1/CT-7 and potentially thereby determine the quality of the resulting immune responses.

In addition, our results indicate that patients with anti-MAGE humoral immunity had worse prognosis. These findings do not necessarily imply that anti-MAGE immune responses directly impair treatment outcomes in ovarian cancer patients. Since antigen density presented by antigen-presenting cells *in vivo* differentially affects the generation of anti-tumor humoral and T cell responses [42], we propose that patients who developed spontaneous immune responses are those with high antigen density because of advanced disease burden, and therefore with worse prognosis. These patients are still likely to benefit from MAGE-directed immunotherapy because their on-going anti-MAGE-A immune responses are not effective. In a previous study of spontaneous humoral immune responses against the NY-ESO-1 CT antigen, the impact of humoral immunity was neutral on patient prognosis [43].

Because the expression of MAGE antigens is regulated by epigenetic mechanisms such as methylation and histone acetylation, we reasoned that MAGE antigen expression in tumors may be the result of the activation of a coordinated gene-expression program, rather than a series of independent events [6]. Using analytical methods, the present study identified significant co-expression among the MAGE antigens. These results support the notion that ovarian cancer acquires a gametogenic transcription profile, in which typically silenced genes are now activated leading to tumor progression. Our results indicate that MAGE-A4 is the central gene that directs the pattern of expression of the other genes, with MAGE-C1 only emerging sporadically in the phylogenetic tree. Taken together, our results suggest that MAGE-A1, -A10 and -C1 are possible prognostic factors in ovarian cancer, with MAGE-A1 and A-10 associated with poor prognosis; and MAGE-C1/CT7 associated with improved prognosis. We propose MAGE-A1 and MAGE-A10 as priority targets for immunotherapy in ovarian cancer. Since MAGE-A4 exhibits a relatively high frequency of expression, and appears to direct a major pattern of co-expression of other MAGE antigens (Fig. 2b), we also propose MAGE-A4 as a priority target for ovarian cancer immunotherapy.

## Supporting Information

Table S1 Primers for MAGE genes for rt-PCR.
(DOCX)

Table S2 MAGE antibodies and conditions for IHC.
(DOCX)

Table S3 MAGE antigen status in ovarian cancer.
(DOCX)
