# Supplementary material for: Expression and Immune Responses to MAGE Antigens Predict Survival in Epithelial Ovarian Cancer
Source: PLoS One. 2014 Aug 7;9(8):e104099. doi: 10.1371/journal.pone.0104099 (PMC4125181; doi:10.1371/journal.pone.0104099)
Supplement: Table S1 — Primers for MAGE genes for rt-PCR. (DOCX) [file pone.0104099.s001.docx]

**Supplemental Table 1: Primers for MAGE genes for rt-PCR**

| **Gene** | **Primer** | **Sequence** | **Amplicon Length^a^** |
| --- | --- | --- | --- |
| MAGE-A1 | Specific Sense | 5’- GCT GGA ACC CTC ACT GGG TTG CC -3’ | 421 |
|  | Anti-Sense | 5’- CGG CCG AAG GAA CCT GAC CCA G -3’ |  |
| MAGE-A3 | Specific Sense | 5’- GAA GCC GGC CCA GGC TCG -3’ | 423 |
|  | Anti-Sense | 5’- GGA GTC CTC ATA GGA TTG GCT -3’ |  |
| MAGE-A4 | Specific Sense | 5’- GAG CAG ACA GGC CAA CCG -3’ | 446 |
|  | Anti-Sense | 5’- AAG GAC TCT GCG TCA GGC -3’ |  |
| MAGE-A10 | Specific Sense | 5’- GGA ACC CCT CTT TTC TAC AGA C -3’ | 502 and 411 |
|  | Anti-Sense | 5’- TCC TCT GGG GTG CTT GGT ATT A -3’ |  |
| MAGE-C1 | Specific Sense | 5’- GAC GAG GAT CGT CTC AGG TCA GC -3’ | 632 |
|  | Anti-Sense | 5’- ACA GGT CGC CTG TCT TCC TA -3’ |  |
| GAPDH | Specific Sense | 5'- TCT TCA CCA CCA TGG AGA AG-3' | 204 |
|  | Anti-Sense | 5'- CAA AGT TGT CAT GGA TGA CCT TGG-3' |  |

^a^ According to the sequences listed in GenBank
