# Supplementary material for: Expression and Immune Responses to MAGE Antigens Predict Survival in Epithelial Ovarian Cancer
Source: PLoS One. 2014 Aug 7;9(8):e104099. doi: 10.1371/journal.pone.0104099 (PMC4125181; doi:10.1371/journal.pone.0104099)
Supplement: Table S2 — MAGE antibodies and conditions for IHC. (DOCX) [file pone.0104099.s002.docx]

**Supplemental Table 2: MAGE antibodies and conditions for IHC**

| **Antibody** | **Clone** | **Pretreatment** | **Dilution** | **Staining Time**  **(minutes)** | **3,3’-diaminobenzidine**  **(DAB)+**  **(minutes)** |
| --- | --- | --- | --- | --- | --- |
| **MAGE-A3** | LS-B4662  Polyclonal | Citrate buffer  pH 6.0 | 1:200 | 60 | 5 |
| **MAGE-A4** | 57b  Monoclonal | Citrate buffer  pH 6.0 | 1:10 | 30 | 5 |
| **MAGE-A10** | A3  Monoclonal | pH target retrieval solution | 2:1 | Overnight | 10 |
