# Supplementary material for: Expression and Immune Responses to MAGE Antigens Predict Survival in Epithelial Ovarian Cancer
Source: PLoS One. 2014 Aug 7;9(8):e104099. doi: 10.1371/journal.pone.0104099 (PMC4125181; doi:10.1371/journal.pone.0104099)
Supplement: Table S3 — MAGE antigen status in ovarian cancer. (DOCX) [file pone.0104099.s003.docx]

**Supplemental Table 3: MAGE antigen status in ovarian cancer**

| **CT Antigen Status** | **rt-PCR** | **IHC** | **Correlation** |
| --- | --- | --- | --- |
|  | n = 305 | n = 304 |  |
| **MAGE-A1** | 42 / 281 (15%) | -- | -- |
| **MAGE-A3** | 60 / 283 (21%) | 78 / 286 (27%) | -0.02 (p=0.773) |
| **MAGE-A4** | 114 / 289 (39%) | 106 / 294 (36%) | 0.31 (p<0.001) |
| **MAGE-A10** | 69 / 274 (25%) | 157 / 296 (53%) | 0.14 (p=0.048) |
| **MAGE-C1** | 42 / 267 (16%) | -- | -- |
| **Any MAGE-A** | 174 / 297 (59%) | 218 / 297 (73%) | -- |

A total of 305 patients were studied for MAGE expression by rt-PCR. A total of 304 patients were studied for MAGE expression by IHC. The numerator represents the number of antigen positive tumors or serology. The denominator represents the total number of successful assays for each antigen. Antigen specific numbers vary due to assay viability. Percentages represent the frequency of MAGE expression.
